# The Role of Artificial Intelligence in Endocrine Management: Assessing ChatGPT’s Responses to Prolactinoma Queries

**DOI:** 10.3390/jpm14040330

**Published:** 2024-03-22

**Authors:** Mustafa Can Şenoymak, Nuriye Hale Erbatur, İrem Şenoymak, Sevde Nur Fırat

**Affiliations:** 1 Department of Endocrinology and Metabolism, University of Health Sciences Sultan, Abdulhamid Han Training and Research Hospital, Istanbul 34668, Turkey; 2 Family Medicine Department, Usküdar State Hospital, Istanbul 34662, Turkey; 3 Department of Endocrinology and Metabolism, University of Health Sciences, Ankara Training and Research Hospital, Ankara 06230, Turkey

**Keywords:** artificial intelligence, ChatGPT, prolactinoma, hyperprolactinemia, health literacy

## Abstract

This research investigates the utility of Chat Generative Pre-trained Transformer (ChatGPT) in addressing patient inquiries related to hyperprolactinemia and prolactinoma. A set of 46 commonly asked questions from patients with prolactinoma were presented to ChatGPT and responses were evaluated for accuracy with a 6-point Likert scale (1: completely inaccurate to 6: completely accurate) and adequacy with a 5-point Likert scale (1: completely inadequate to 5: completely adequate). Two independent endocrinologists assessed the responses, based on international guidelines. Questions were categorized into groups including general information, diagnostic process, treatment process, follow-up, and pregnancy period. The median accuracy score was 6.0 (IQR, 5.4–6.0), and the adequacy score was 4.5 (IQR, 3.5–5.0). The lowest accuracy and adequacy score assigned by both evaluators was two. Significant agreement was observed between the evaluators, demonstrated by a weighted κ of 0.68 (*p* = 0.08) for accuracy and a κ of 0.66 (*p* = 0.04) for adequacy. The Kruskal–Wallis tests revealed statistically significant differences among the groups for accuracy (*p* = 0.005) and adequacy (*p* = 0.023). The pregnancy period group had the lowest accuracy score and both pregnancy period and follow-up groups had the lowest adequacy score. In conclusion, ChatGPT demonstrated commendable responses in addressing prolactinoma queries; however, certain limitations were observed, particularly in providing accurate information related to the pregnancy period, emphasizing the need for refining its capabilities in medical contexts.

## 1. Introduction

Hyperprolactinemia is a clinical condition frequently encountered in endocrine practice, characterized by elevated serum prolactin levels, often posing challenges in terms of differential diagnosis and management [1]. While prolactinoma is a significant factor, the multitude of conditions leading to elevated prolactin levels, coupled with the requirement for a multidisciplinary approach due to prolactinoma’s impact on various systems, gives rise to numerous questions and uncertainties among patients [2]. In these situations, patients commonly seek or are referred to endocrinology clinics, directing their inquiries to experts in the field. Responding to patients’ questions about their conditions serves to alleviate unnecessary anxiety and expenses, while also streamlining disease management and improving treatment outcomes [3].

In the current context, artificial intelligence (AI) applications have become ubiquitous and easily accessible repositories of information. Artificial intelligence programs are widely used by both healthcare professionals and patients. As observed from these studies, it has been noted that patients find numerous advantages in the use of such applications. These advantages for patients include gaining a deeper understanding of their medical conditions through research conducted in artificial intelligence programs, as well as engaging in interactive conversations by posing questions in a natural conversational manner, which they might not have had the opportunity to ask their doctors. Additionally, patients benefit from effectively managing their treatment through features like drug reminders, self-monitoring, dosage guidance, and accessing information on the potential side effects of therapy [4,5,6]. On the other hand, healthcare professionals benefit from utilizing artificial intelligence in the process of diagnostic radiology and medical education, as well as in the planning, execution, and composition of medical research within the field [7,8]. Despite the escalating frequency of its application, artificial intelligence in these domains is subject to notable limitations. The occasional lack of up-to-date information, variability in responses to different types of queries, and non-repeatability, resulting in varying responses at different times, can create misleading situations for patients. Additionally, it can be noted among its shortcomings that artificial intelligence in diagnostic processes may introduce bias, exhibit high error rates, and contribute to uniformity in research procedures [9,10].

The responses provided by an artificial intelligence program, Chat Generative Pre-trained Transformer (ChatGPT), to questions posed by patients in various fields have been extensively investigated in numerous studies [11,12,13]. Although results may vary, it has been observed in these studies that ChatGPT generally provides responses with high accuracy, regarding the relevant medical conditions. However, the use of artificial intelligence in prolactinoma has not been studied to date. Our study seeks to assess the adequacy and accuracy of responses generated by ChatGPT in addressing the most prevalent inquiries posed by patients with hyperprolactinemia and prolactinoma attending an endocrinology and metabolism department.

## 2. Materials and Methods

This research identified the 46 most frequently asked questions posed by patients seeking medical attention for hyperprolactinemia at an endocrinology and metabolism department. After compiling the list of questions, they were systematically presented to the artificial intelligence program ChatGPT (GPT3.5, version dated 13 January 2024) via https://chat.openai.com accessed on 13 January 2024. The recorded answers were then documented for subsequent analysis. In order to evaluate its reproducibility, each question was posed to ChatGPT twice.

The responses to inquiries were evaluated by two independent expert physicians working in the department of endocrinology and metabolism. The first reviewer is affiliated with the Department of Endocrinology and Metabolism at the University of Health Sciences, Ankara Training and Research Hospital and the second reviewer is affiliated with the Department of Endocrinology and Metabolism at the University of Health Sciences, İstanbul Sultan Abdülhamid Han Training and Research Hospital. Following this assessment, the responses were scored based on accuracy and adequacy, according to the international guidelines “Diagnosis and Treatment of Hyperprolactinemia: An Endocrine Society Clinical Practice Guideline” and “Guidelines of the Pituitary Society for the diagnosis and management of prolactinomas” [14,15,16]. The accuracy scale was operationalized as a 6-point Likert scale, with 1 denoting complete inaccuracy, 2 indicating a greater degree of inaccuracy than accuracy, 3 representing an approximate balance between accuracy and inaccuracy, 4 suggesting a higher accuracy level than inaccuracy, 5 reflecting near complete accuracy, and 6 signifying complete accuracy). Likewise, the adequacy scale was structured as a 5-point Likert scale, where 1 denoted complete inadequacy, 2 represented a greater inadequacy compared to adequacy, 3 signified an approximate equilibrium between adequacy and inadequacy, 4 indicated a higher degree of adequacy than inadequacy, and 5 indicated complete adequacy.

### Statistical Analyses

Data analyses were conducted using IBM SPSS Statistics version 25.0 software. Outcome scores were presented descriptively, encompassing median [interquartile range (IQR)] values and mean [standard deviation (SD)] values. Group-wise comparisons were performed using either the Mann–Whitney U test or the Kruskal–Wallis test (SPSS, version 25.0). Inter-rater concordance was evaluated utilizing the weighted κ statistic, covering a comprehensive range of scores, from 1 to 6 for accuracy and 1 to 5 for adequacy. Statistical significance was established at a threshold of *p*  < 0.05. Significance values were adjusted by the Bonferroni correction for multiple tests. Responses to the repeated queries were subjected to comparison using the Wilcoxon signed rank test in order to assess reproducibility.

## 3. Results

Artificial intelligence was employed to address commonly encountered inquiries regarding prolactinoma posed by patients during routine endocrinology practice. Subsequently, two endocrinology and metabolism experts systematically assessed and assigned scores to the responses provided by ChatGPT (Table 1).

Among the 46 questions under evaluation, the median average accuracy score demonstrated complete accuracy, registering at 6.0 (IQR, 5.4–6). The overall mean (SD) accuracy score of 5.5 (0.9) was positioned within the range spanning near complete accuracy and complete accuracy (Table 2).

Regarding adequacy, the average median score reached 4.5 (IQR, 3.5–5.0), indicating a level situated between a higher degree of adequacy than inadequacy and complete adequacy. The mean (SD) adequacy score of 4.2 (0.9) further substantiates this finding (Table 3).

According to the reproducibility test, the responses to the original and repeated questions did not differ significantly in terms of both accuracy and adequacy (with respective *p* values of 0.79 and 0.24, determined using the Wilcoxon signed rank test). The responses to repeated questions garnered a median accuracy score of 6 (IQR, 5.5–6.0; mean [SD] score, 5.5 [0.7]) and a median adequacy score of 4.5 (IQR, 4.0–5.0; mean [SD] score, 4.4 [0.7]). Despite variations in sentence structure and minimal changes, no major alterations in content were identified across the responses.

Evaluators demonstrated concordance, as evidenced by a weighted κ of 0.68 (*p* = 0.08) for accuracy and a substantial agreement reflected by a weighted κ of 0.66 (*p* = 0.04) for adequacy.

Questions were stratified into distinct thematic categories, encompassing general information, diagnostic process, treatment process, follow-up, and the pregnancy period. The average median accuracy scores for these categories were 6.0 (IQR, 6.0–6.0), 6.0 (IQR, 6.0–6.0), 6.0 (IQR, 5.0–6.0), 5.0 (IQR, 5.0–6.0), and 5.0 (IQR, 2.5–5.7), respectively. The corresponding average mean [SD] scores were 5.9 [0.2], 5.9 [0.2], 5.7 [0.4], 5.5 [0.5], and 4.4 [1.6], respectively. Statistical analysis using the Kruskal–Wallis test (*p* = 0.005) indicated significant differences among these thematic groups. The subsequent pairwise post hoc Dunn test, employing Bonferroni adjustments, revealed significant distinctions, specifically for general information questions vs. pregnancy period questions (*p* = 0.009) and diagnostic process questions vs. pregnancy period questions (*p* < 0.012) (Table 2).

**Figure 1:**
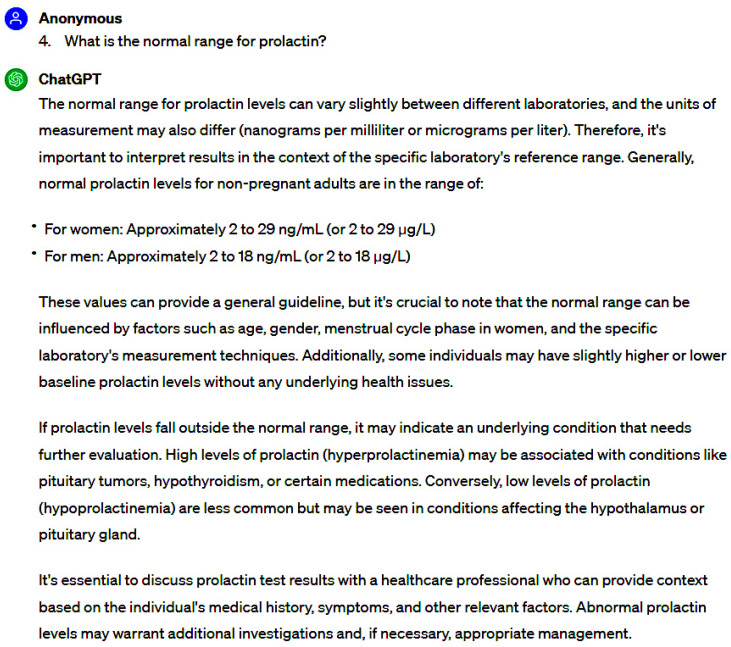
Samples of responses with the highest accuracy scores.

**Figure 2:**
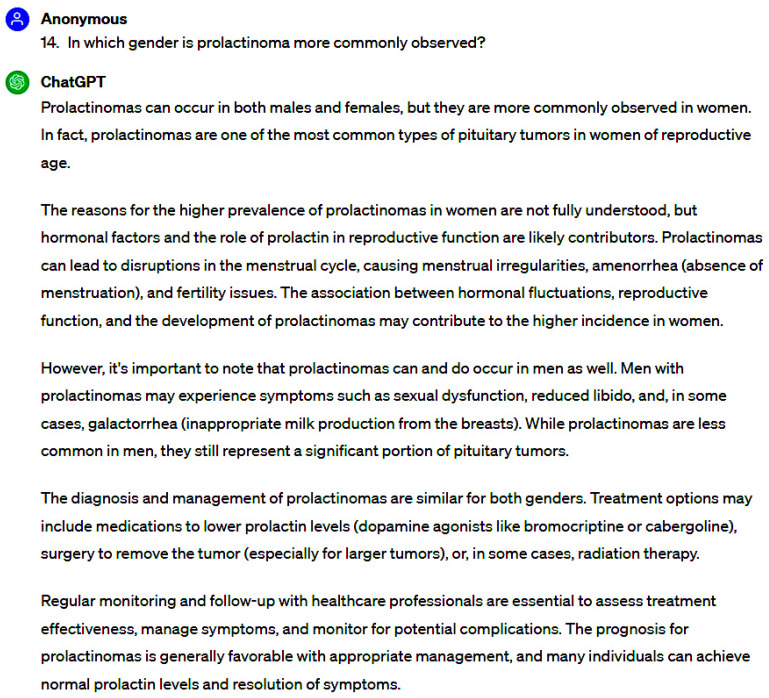
Samples of responses with the highest accuracy scores.

**Figure 3:**
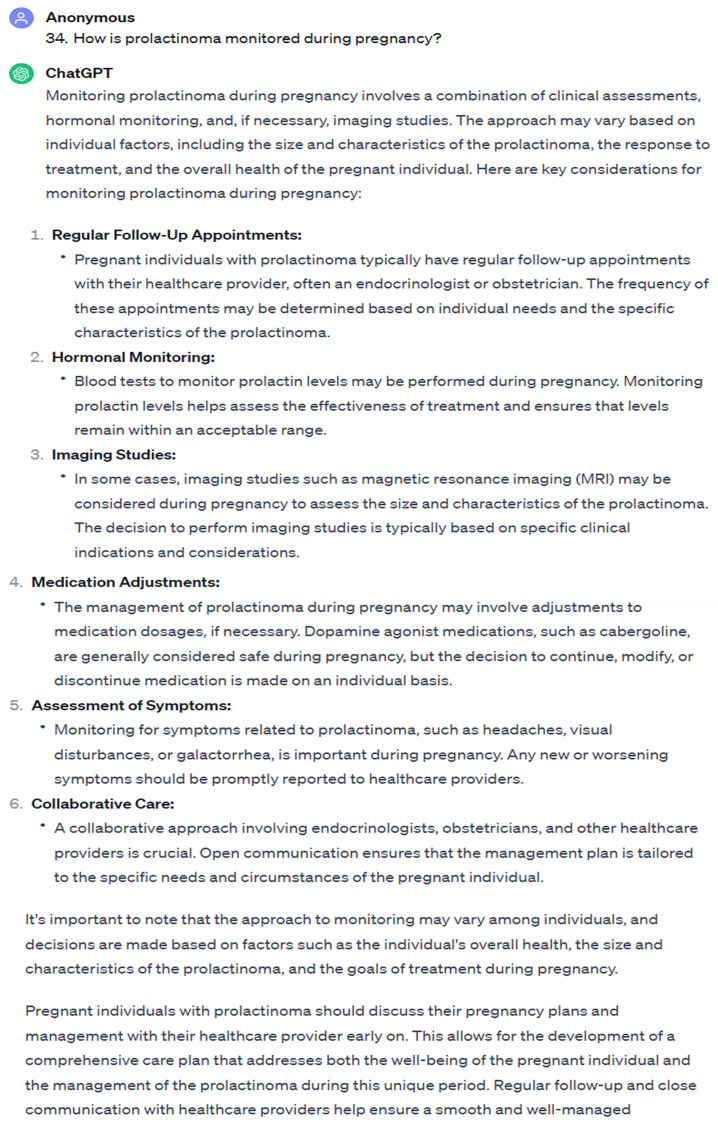
First answer with the lowest accuracy score.

**Figure 4:**
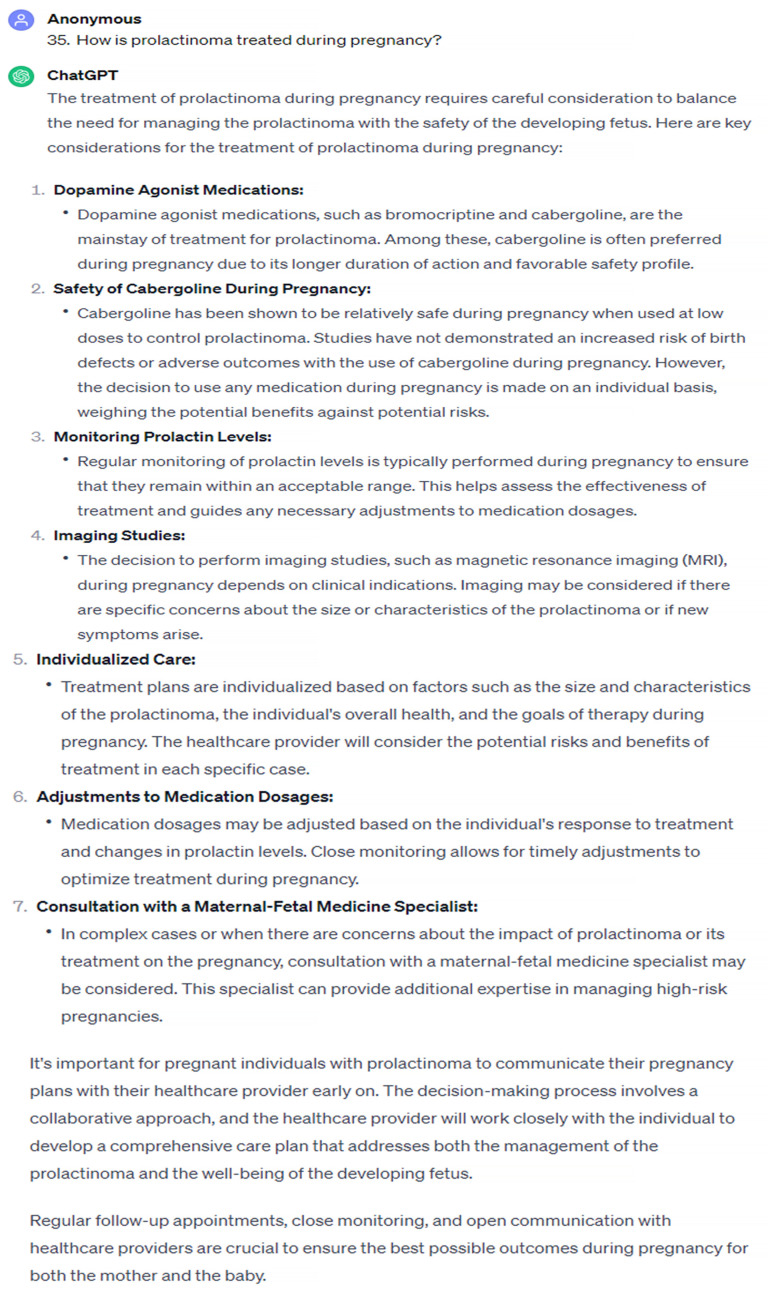
Second answer with the lowest accuracy score.

Evaluator #1 attributed the highest accuracy score (6.0) to 35 questions, constituting 76.1% of the total, while 8 questions (17.4%) garnered a rating of nearly completely accurate (2.0). Conversely, evaluator #2 bestowed the highest accuracy score (6.0) upon 28 questions (60.9%), with 15 questions (32.6%) characterized as nearly completely accurate (2.0). Both assessors assigned the lowest accuracy score (2.0) to the same two questions, signifying a notable disparity towards inaccuracy rather than accuracy (Figure 1, Figure 2, Figure 3 and Figure 4).

Evaluator #1 assessed the responses to 21 questions (45.7%) as entirely adequate, 13 (28.3%) as demonstrating a higher degree of adequacy than inadequacy, and 10 (21.7%) as approximately equal in terms of adequacy and inadequacy. For evaluator #2, the answers to 24 questions (52.2%) received a rating of complete adequacy, 10 (21.7%) were deemed to have a higher degree of adequacy than inadequacy, and 11 (23.9%) were evaluated as approximately equal in terms of adequacy and inadequacy. Both evaluators assigned the lowest accuracy score of 2 and, notably, none of the questions received a score indicating complete inadequacy.

A compelling correlation between accuracy and adequacy was demonstrated, with a Spearman correlation coefficient (r) of 0.64 (*p* < 0.001), observed for all queried questions.

Similarly, when questions were categorized into distinct themes, including general information, diagnostic process, treatment process, follow-up, and the pregnancy period, the average median adequacy scores were 5.0 (IQR, 4.2–5.0), 5.0 (IQR, 4.0–5.0), 4.5 (IQR, 3.5–5.0), 3.5 (IQR, 2.7–4.5), and 3.5 (IQR, 3.0–4.8), respectively. The corresponding average mean [SD] scores were 4.9 [1.1], 4.5 [0.8], 4.3 [0.8], 3.5 [1.1], and 3.8 [0.8], respectively. While the Kruskal–Wallis test (*p* = 0.023) indicated statistically significant differences among the thematic groups, post hoc pairwise analysis did not reveal any significant distinctions (Table 3).

## 4. Discussion

This study marks a pioneering exploration into the utilization of an artificial intelligence tool, ChatGPT, for addressing inquiries related to hyperprolactinemia and prolactinoma. To the best of our knowledge, it serves as the first comprehensive study systematically examining responses generated by ChatGPT, specifically within the realm of prolactinoma. The evaluation of the effectiveness of artificial intelligence in endocrinological patient communication through the two crucial dimensions of accuracy and adequacy revealed noteworthy findings.

In contemporary medical practices, a crucial aspect of successful disease management involves patients being aware of their conditions and actively participating in the treatment and management process. Studies have indicated a positive association between increased health literacy and improved disease outcomes [17,18]. Therefore, various approaches are being explored to enhance health literacy. Technology, particularly internet-based tools, is increasingly replacing traditional educational and informational resources to promote health literacy [19]. The emergence of artificial intelligence, notably as the most accessible and popular among these technologies, underscores its widespread use in various fields. In our study, we utilized ChatGPT as an artificial intelligence tool, which is the most frequently used and popular, and we investigated its role in the management of prolactinoma.

Prolactinoma is a commonly encountered condition in endocrinology practice, characterized by several pitfalls in diagnosis and management. The rationale behind selecting prolactinoma for this study stems from the need for patients to obtain information in this confusing disease, which necessitates a thorough evaluation and is commonly accompanied by a multitude of inquiries. Hence, there is a necessity for a supportive tool to assist patients in acquiring knowledge. In our study, the questions were compiled by actively practicing endocrinologists and consisted of inquiries commonly posed by patients in the outpatient clinic setting. The content and techniques of the questions were structured to mimic those typically encountered in clinical practice (Table 1). This approach enhances the alignment of our study with real-life data, ultimately bridging real-world data with an artificial intelligence platform.

In our study, it was found that ChatGPT demonstrated a commendable performance in offering accurate and adequate responses to the questions posed by patients seeking information on hyperprolactinemia and prolactinoma. Upon subjecting it to group analysis, specific areas of concern became apparent and, notably, a lower accuracy in information related to pregnancy was revealed. According to the Endocrine Society Clinical practice guideline and guidelines of the Pituitary Society for the diagnosis and management of prolactinomas, in prolactinoma cases during pregnancy, it is generally recommended to discontinue dopamine agonist therapy and ensure close monitoring, aimed at minimizing fetal exposure to the medication. However, exceptions exist for cases presenting with symptoms indicative of mass effect or those involving macroprolactinomas. The routine monitoring of prolactin levels is discouraged during pregnancy, due to the inherent ambiguity in interpreting such levels in pregnant individuals. Instead, emphasis is placed on regular clinical evaluations and the assessment of visual fields as part of the follow-up regimen for optimal management [14,15,16]. Despite these established guidelines, our study highlights that ChatGPT’s responses on this topic, particularly those suggesting prolactin measurement and continuing medical treatment during pregnancy, can be misleading for patients (Figure 3 and Figure 4). The observed misguidance in ChatGPT’s responses underscores the importance of the cautious interpretation of AI-generated information in medical contexts. It emphasizes the need for the ongoing refinement and validation of AI models to align more closely with evolving clinical knowledge and guidelines. Additionally, this finding serves as a reminder of the critical role that human expertise plays in interpreting AI-generated information, especially in complex medical scenarios where context and individual patient factors are paramount.

While there may be a limited body of research specifically addressing prolactinoma, the effectiveness of artificial intelligence in addressing patient queries has been investigated in numerous studies spanning various diseases and medical conditions [11,12,13,20,21]. In a study conducted by Goodman and colleagues, 33 physicians from different medical specialties posed 284 questions to ChatGPT and the responses were evaluated for accuracy and completeness, similar to our study. The accuracy of the responses was assessed between almost completely and completely correct, and completeness was evaluated as being complete and comprehensive [20]. In another study related to bariatric surgery, ChatGPT was found to answer 86.8% of the questions; however, unlike our study, proficiency and accuracy were not separately evaluated and a common scoring system was employed [22].

Studies on the success of artificial intelligence programs yield conflicting results. While many studies report successful outcomes, some have resulted in failure. For instance, in a study conducted by Rahsepar and colleagues, it was noted that ChatGPT provided correct answers to 70% of 120 questions related to lung cancer but faced criticism for not achieving complete accuracy. Similarly, questions pertaining to hepatocellular carcinoma and cirrhosis were answered with over 70% accuracy, but the comprehensiveness remained at around 40%, indicating a need for improvement [12]. Recent research has compared the performance of ChatGPT’s old and new versions in responding to medical queries, revealing that the use of newer versions has increased the success rate [23].

A primary constraint within our investigation, as well as a significant quandary concerning artificial intelligence systems, pertains to the variability in responses contingent upon question formulation. This variability, induced by discrepancies in patients’ articulatory skills when formulating inquiries, could yield divergent responses. Additionally, despite the appraisal of responses by healthcare experts, uncertainties persist regarding whether these responses will exert an analogous impact on the patients. Consequently, patients may not fully avail themselves of precise and comprehensive information, as observed within such investigations. To alleviate this challenge, multicenter studies should be conducted, involving patients with diverse sociocultural backgrounds, educational proficiencies, and linguistic diversities, wherein the data evaluated by healthcare professionals should also be concurrently assessed by patients.

Some other constraints impact the robustness of our study. Firstly, ChatGPT’s reliance on information up to 2022 raises concerns about its currency and the potential incorporation of outdated knowledge. Secondly, the subjective nature of adequacy evaluations introduces potential bias, as responses were not objectively measured in terms of appropriateness. Additionally, the evaluation solely focused on ChatGPT, overlooking the various other artificial intelligence platforms available for use in the medical field. Comparative evaluations of these platforms could significantly enhance the study, enabling patients to find the most appropriate platform for their healthcare needs.

## 5. Conclusions

This study underscores the commendable performance of ChatGPT in addressing patient inquiries related to hyperprolactinemia and prolactinoma and highlights the positive potential of artificial intelligence in enhancing patient communication and promoting health literacy. However, the findings also reveal specific areas of concern, particularly in the realm of pregnancy-related information, where ChatGPT exhibited a lower accuracy. This highlights the critical need for the careful interpretation of artificial intelligence-generated information in intricate medical situations, emphasizing the importance of the continuous refinement and validation of AI models, as well as the pivotal role of human expertise.

## Figures and Tables

**Table 1:** Questions and average accuracy and adequacy scores given by medical doctors to the AI’s responses ^a^.

Questions	Accuracy Score ^b^	Adequacy Score ^c^
What is prolactin?	6	5
What is the physiological role of prolactin in the body?	6	5
How is prolactin measured?	6	3
What is the normal range for prolactin?	6	5
What precautions should be taken before blood sampling for prolactin measurement?	5.5	4
What factors contribute to elevated levels of prolactin?	6	5
Which medications can increase prolactin levels?	6	5
What are the symptoms of hyperprolactinemia?	6	4.5
What is macroprolactinemia?	6	4
What is prolactinoma?	6	5
What are the symptoms of prolactinoma?	6	4
How is the diagnosis of prolactinoma established?	5.5	3
In which gender is prolactinoma more commonly observed?	6	5
How can it be determined whether the elevated prolactin is due to a pituitary adenoma?	6	4
What is macroprolactinoma?	6	5
What is the treatment for prolactinoma?	6	5
Should every patient be treated, or is it possible to opt for a conservative approach with regular monitoring without intervention?	5	4.5
How is cabergoline used in the treatment of prolactinoma?	6	5
What are the side effects of cabergoline?	6	3
How is bromocriptine used in the treatment of prolactinoma?	5.5	4
What are the side effects of bromocriptine?	5	3
In what situations is surgery required for prolactinoma?	6	5
Is radiotherapy administered in the treatment of prolactinoma?	6	5
What should be the target prolactin level with treatment?	5	4.5
How long should medication be administered in the treatment of prolactinoma?	6	3
Do symptoms completely resolve after treatment?	5.5	3
Can prolactinoma shrink with medical treatment?	6	5
Is it possible to achieve complete recovery from prolactinoma after treatment?	6	4
How often should a patient diagnosed with prolactinoma consult a doctor?	5	2
How often should an MRI be performed in prolactinoma cases?	5	2.5
Can there be a recurrence after discontinuing medication for prolactinoma?	5.5	3.5
Can patients with prolactinoma conceive?	5	3
How is prolactinoma monitored during pregnancy?	2	3
How is prolactinoma treated during pregnancy?	2	3
In a pregnant patient with prolactinoma, when should medical treatment be discontinued?	5	3.5
Can women with prolactinoma use medication after childbirth?	5	3.5
Is breastfeeding allowed for a patient with prolactinoma after childbirth?	5	4
Should postmenopausal prolactinoma be treated?	5.5	4
What are the complications of elevated prolactin levels?	6	4.5
Which other hormones are affected by hyperprolactinemia?	5.5	5
What ocular manifestations are associated with prolactinoma?	6	5
Does prolactinoma cause headaches?	6	5
Does elevated prolactin affect sexual function in men?	6	5
Does hyperprolactinemia cause menstrual irregularities?	6	5
Is it possible to conceive while having elevated prolactin levels?	6	5

^a^ AI indicates artificial intelligence. ^b^ The accuracy scale was operationalized as a 6-point Likert scale, with 1 denoting complete inaccuracy, 2 indicating a greater degree of inaccuracy than accuracy, 3 representing an approximate balance between accuracy and inaccuracy, 4 suggesting a higher accuracy level than inaccuracy, 5 reflecting near complete accuracy, and 6 signifying complete accuracy. ^c^ The adequacy scale was structured as a 5-point Likert scale, where 1 denoted complete inadequacy, 2 represented a greater inadequacy compared to adequacy, 3 signified an approximate equilibrium between adequacy and inadequacy, 4 indicated a higher degree of adequacy than inadequacy, and 5 indicated complete adequacy.

**Table 2:** Assessing accuracy ^a^ and comparing responses generated by AI through categorization into distinct question groups.

	General Information	Diagnostic Process	Treatment Process	Follow-Up	Pregnancy Period	Total	*p* Value
Rater 1							
Median (IQR)	6.0 (6.0–6.0)	6.0 (6.0–6.0)	6.0 (5.5–6.0)	6.0 (5.0–6.0)	5.0 (2.5–5.7)	6.0 (5.8–6.0)	0.001
Mean (SD)	6.0 (0.0)	6.0 (0.0)	5.8 (0.4)	5.7 (0.5)	4.4 (1.6)	5.6 (0.9)	
Rater 2							
Median (IQR)	6.0 (6.0–6.0)	6.0 (6.0–6.0)	6.0 (5.0–6.0)	5.0 (5.0–6.0)	5.0 (2.5–5.7)	6.0 (5.0–6.0)	0.015
Mean (SD)	5.8 (0.3)	5.8 (0.4)	5.5 (0.5)	5.4 (0.5)	4.4 (1.6)	5.5 (0.9)	
Average Score							
Median (IQR)	6.0 (6.0–6.0)	6.0 (6.0–6.0)	6.0 (5.2–6.0)	5.5 (5.0–6.0)	5.0 (2.5–5.7)	6.0 (5.4–6.0)	0.005
Mean (SD)	5.9 (0.2)	5.9 (0.2)	5.7 (0.4)	5.5 (0.5)	4.4 (1.6)	5.5 (0.9)	

^a^ The accuracy scale was operationalized as a 6-point Likert scale, with 1 denoting complete inaccuracy, 2 indicating a greater degree of inaccuracy than accuracy, 3 representing an approximate balance between accuracy and inaccuracy, 4 suggesting a higher accuracy level than inaccuracy, 5 reflecting near complete accuracy, and 6 signifying complete accuracy. Kruskal–Wallis (>2 variables) tests were used for non-parametric variables and data were given as median [interquartile range (IQR)] values and mean [standard deviation (SD)]. *p* values less than 0.05 were considered statistically significant. Significance values were adjusted using the Bonferroni correction for multiple tests.

**Table 3:** Scoring adequacy ^a^ and comparing responses generated by artificial intelligence based on categorization into question groups.

	General İnformation	Diagnostic Process	Treatment Process	Follow-Up	Pregnancy Period	Total	*p* Value
Rater 1							
Median (IQR)	5.0 (4.5–5.0)	5.0 (4.0–5.0)	5.0 (3.5–5.0)	4.0 (3.0–4.5)	3.0 (3.0–4.8)	5.0 (3.0–5.0)	0.018
Mean (SD)	4.8 (0.4)	4.6 (0.8)	4.4 (0.9)	3.6 (1.0)	3.6 (0.9)	4.3 (0.9)	
Rater2							
Median (IQR)	5.0 (4.0–5.0)	5.0 (4.0–5.0)	4.0 (3.5–5.0)	3.0 (2.5–4.5)	4.0 (3.0–4.8)	4.0 (3.0–5.0)	0.059
Mean (SD)	4.7 (0.5)	4.4 (0.8)	4.2 (0.8)	3.0 (1.1)	3.9 (0.8)	4.1 (0.9)	
Average Score							
Median (IQR)	5.0 (4.2–5.0)	5.0 (4.0–5.0)	4.5 (3.5–5.0)	3.5 (2.7–4.5)	3.5 (3.0–4.8)	4.5 (3.5–5.0)	0.023
Mean (SD)	4.9 (1.1)	4.5 (0.8)	4.3 (0.8)	3.5 (1.1)	3.8 (0.8)	4.2 (0.9)	

^a^ The adequacy scale was structured as a 5-point Likert scale, where 1 denoted complete inadequacy, 2 represented a greater inadequacy compared to adequacy, 3 signified an approximate equilibrium between adequacy and inadequacy, 4 indicated a higher degree of adequacy than inadequacy, and 5 indicated complete adequacy. Kruskal–Wallis (>2 variables) tests were used for non-parametric variables and data were given as median [interquartile range (IQR)] values and mean [standard deviation (SD)]. *p* values less than 0.05 were considered statistically significant. Significance values were adjusted using the Bonferroni correction for multiple tests.

## Data Availability

The data supporting the findings of this study are available upon reasonable request to the corresponding author.
